# Edaphic and climatic factors influence on the distribution of soil transmitted helminths in Kogi East, Nigeria

**DOI:** 10.1038/s41598-021-88020-1

**Published:** 2021-04-19

**Authors:** Clement Ameh Yaro, Ezekiel Kogi, Sodangi Abdulkarim Luka, Mohamed A. Nassan, Junaidu Kabir, Kenneth Nnamdi Opara, Helal F. Hetta, Gaber El-Saber Batiha

**Affiliations:** 1 Department of Animal and Environmental Biology, University of Uyo, Uyo, Akwa Ibom State Nigeria; 2 Department of Zoology, Ahmadu Bello University, Zaria, Nigeria; 3 Department of Clinical Laboratory Sciences, Turabah University College, Taif University, P.O. Box 11099, Taif, 21944 Saudi Arabia; 4 Department of Veterinary Public Health and Preventive Medicine, Ahmadu University, Zaria, Nigeria; 5 Department of Medical Microbiology and Immunology, Faculty of Medicine, Assiut University, Assiut, 71515 Egypt; 6 Department of Pharmacology and Therapeutics, Faculty of Veterinary Medicine, Damanhour University, Damanhour, AlBeheira 22511 Egypt

**Keywords:** Ecological epidemiology, Ecological genetics

## Abstract

The need for a reliable risk map in the control of soil-transmitted helminths (STHs) in Kogi East, North Central Nigeria is very important. This study was carried out to determine the effect of environmental risk factors on geospatial distribution of STHs. Epidemiological data were obtained from a district-wide survey conducted in 2018 in Kogi East. Edaphic and climatic factors were downloaded as spatial layers from international recognised health data resources centres. A total of 24 environmental factors were used in determining the risk map of STHs using MaxEnt tool. The predicted high-risk areas of *A. lumbricoides*, hookworms and *S. stercoralis* were the central part of Kogi East covering parts of Dekina, Ofu, Igalamela-Odolu, Olamaboro and Omala LGAs with probability of 0.8 to 1.00. Among the factors investigated; Temperature [mean diurnal temperature range (BIO2), temperature annual range (BIO7) and maximum temperature of the warmest month (BIO5)], precipitation [precipitation of the wettest quarter (BIO16)], and soil clay contents were the five factors that exerted most significant influence on the geospatial distribution of STHs in Kogi East, Nigeria. Public health control programmes on STHs should target high-risk areas by including them in mass drug administration, health education as well as provision of water, sanitation and hygiene infrastructures.

## Introduction

Soil-transmitted helminths (STHs) are one of the leading causes of global health problems especially in poorest and deprived communities where implementation of control measures are difficult to maintain. It is caused by parasitic nematodes such as *Ascaris lumbricoides*, hookworms and *Trichuris trichiura* which are transmitted through contact with parasites eggs or larvae^1^. These parasites are responsible for more than 40% of worldwide morbidity from tropical infections^2^. About 2 billion people are infected with STHs worldwide^3^.

Soil-transmitted helminthiasis (STH) is the most prevalent Neglected Tropical Diseases (NTDs) in Nigeria^4^. Infections with STH contributes to malnutrition, iron-deficiency and anaemia and can have adverse effect on physical and mental growth in childhood^5–7^. In Nigeria, the World Health Organization (WHO) through the Federal Ministry of Health carried out large-scale distribution of anthelminthic drugs in many parts of the country without prior diagnosis and identification of high risk areas^8,11^, leading to uneven distribution of drugs.

The use of Geographic Information Systems (GIS) and Remote Sensing (RS) to better understand helminths distributions and their ecology is highly effective, they serve as decision-making tools for the identification areas of high-risk and hotspots that will help in the design, implementation and monitoring of control programmes^12^. Due to the importance of environmental factors in the transmission processes, it is relevant to establish a relationships between environmental risk factors and spatial patterns of infection. Several studies reported the ecological associations between STH distributions and climatic factors^13–17^. GIS tools have been used successfully to describe the environmental factors associated with patterns of STH infection in selected geographical locations, and has helped to identify the relative importance of different environmental factors in determining geographic distributions^18,23^.

The global access to geospatial health resources and analysis of data with good software’s have enabled the development of unique and low-cost digital health maps and transmission models for tropical diseases. These methods allows visualization of rates of disease transmission and unique way of data presentation that are purely spatial with rich descriptive analysis. They also allows easy combination of disease data with demographic, economic and environment data^24^.

Kogi East, North-Central Nigeria is endemic for STHs but lack adequate information on the environmental risk factors associated with STHs transmission^25^. Therefore, there is need to identify the high-risk areas of STHs using the epidemiological data alongside environmental factors to better understand the ecology and distribution of STHs so as to design an appropriate control strategies. Also, previous studies on species distribution models (SDM) in Nigeria on STHs only considered climatic factors as determinants^26,27^. Edaphic factors which plays an important role in the STH ecology were overlooked. This study therefore included edaphic predictors in determining the distribution of STHs in Kogi East, North Central Nigeria so as to enhance the model, it is the first of its kind in Nigeria.

## Materials and methods

### Study area

Kogi East located in Kogi State, North Central Nigeria. It is a geographical region comprising of nine (9) Local Government Areas (LGAs); Ankpa, Bassa, Dekina, Ibaji, Idah, Igalamela/Odolu, Ofu, Olamaboro and Omala. The region is located between latitude 6º32′33.8′′N to 8º02′44.8′′N and longitude 6º42′08.5′′E to 7º51′50.3′′E. It occupies an area of 26,197 square kilometres sharing boundaries with six (6) states of Nigeria^28^. The population of the region at 2006 is 1,479,144 with a projected population of 1,996,700 at 2016^29^.

### Ethical approval and informed consent

Ethical clearance was obtained from Research Ethics Committee, Kogi State Ministry of Health (KSMoH), Lokoja with reference number MOH/KGS/1376/1/82 and permission was obtained from the State Universal Basic Education Board (SUBEB), Lokoja with reference number KG/SUBEB/GEN/04/’T’ which was conveyed to the Education Secretaries of the 9 LGAs and the Headmasters (mistress) of the schools.

This study follows guidelines for the care and use of human samples established by the Human Care and Use Committee of the Ahmadu Bello University, Zaria, Kaduna State, Nigeria and the Research Ethics Committee, Kogi State Ministry of Health (KSMoH), Lokoja.

### Statement of consent from participants

Written consents were obtained from the guardians/parents of study participants, informing them of their rights and granting permission for their children to participate in the study.

### Source of epidemiological data

The epidemiological data used for this study were obtained from an earlier district-wide survey carried out in 2018 (Table 1)^25^ in rural communities of Kogi East, Kogi State, Nigeria. The study obtained samples from school-children of age 5 to 14 years. Samples collected were examined using formal ether sedimentation technique. The study was carried out in schools that did not receive anthelminthic drugs during the yearly periodic deworming exercise carried out by the State Ministry of Health. During the survey, the geographical coordinates of each school and community were captured within the school premises using a handheld Global Positioning system (GPS) device, Garmin 12XL (Garmin Corp, USA).

**Table 1:** Epidemiological Data from District Wide Survey Conducted in 2018 by Yaro et al. (2020) in Kogi East, North Central Nigeria.

LGAs	Communities (n)	Latitude	Longitude	Number Positive (Prevalence in %)			
				STHs	*A. lumbricoides*	Hookworms	*S. stercoralis*
Ankpa	Ikanekpo (21)	7.4440	7.5398	8 (38.1)	8 (38.1)	0 (0)	0 (0)
	Opulega (25)	7.4146	7.6137	5 (20.0)	0 (0)	5 (20.0)	0 (0)
	Ogodo (37)	7.3964	7.5786	6 (16.2)	1 (2.7)	4 (10.8)	1 (2.7)
	Enokpoli (11)	7.4508	7.6454	1 (9.1)	0 (0)	1 (9.1)	0 (0)
	Enjema (18)	7.4746	7.6599	3 (16.7)	3 (16.7)	0 (0)	0 (0)
Bassa	Akakana (29)	7.9227	7.1777	3 (10.3)	2 (6.9)	1 (3.4)	0 (0)
	Oguma (31)	7.8864	7.0644	6 (19.4)	1 (3.2)	5 (16.1)	0 (0)
	Sheria 1 (36)	7.8920	7.0764	3 (8.3)	1 (2.8)	2 (5.6)	0 (0)
	Sheria 2 (26)	7.8914	7.0798	4 (15.4)	4 (15.4)	0 (0)	0 (0)
	Londu (28)	7.8687	7.0664	6 (21.4)	5 (17.9)	4 (14.3)	0 (0)
Dekina	Olubojo (27)	7.6298	7.0880	7 (25.9)	0 (0)	7 (25.9)	2 (7.4)
	Ojofu (20)	7.5328	7.1806	0 (0)	0 (0)	0 (0)	0 (0)
	Ajiyolo-Akabe (30)	7.5702	7.0976	0 (0)	0 (0)	0 (0)	0 (0)
	Odu-Ogbaloto (35)	7.6138	7.0950	5 (14.3)	2 (5.7)	5 (14.3)	2 (5.7)
	Olofu (31)	7.5358	7.1561	6 (19.4)	0 (0)	6 (19.4)	0 (0)
Ibaji	Itoduma (36)	6.9232	6.6836	2 (5.6)	0 (0)	1 (2.8)	1 (2.8)
	Onyedega (40)	6.8902	6.6755	5 (12.5)	2 (5.0)	3 (7.5)	0 (0)
	Unale (40)	6.9093	6.7167	3 (7.5)	1 (2.5)	1 (2.5)	1 (2.5)
	Ejule-Ojebe (40)	7.0019	6.7278	2 (5.0)	2 (5.0)	0 (0)	0 (0)
	Odogwu (41)	6.9603	6.7288	2 (4.9)	0 (0)	2 (4.9)	0 (0)
Idah	Ukwaja (26)	7.1089	6.7454	11 (42.3)	0 (0)	11 (42.3)	1 (3.8)
	Igalogba (24)	7.1206	6.7476	3 (12.5)	0 (0)	3 (12.5)	0 (0)
	Sabon Gari (21)	7.1091	6.7403	4 (19.0)	0 (0)	4 (19.0)	0 (0)
	Ede (29)	7.1014	6.7386	2 (6.9)	0 (0)	3 (10.3)	1 (3.4)
	Ubomu (24)	7.1252	6.7413	2 (8.3)	0 (0)	2 (8.3)	0 (0)
Igalamela/Odolu	Ogbogbo 1 (29)	7.1059	6.8088	2 (6.9)	0 (0)	1 (3.4)	0 (0)
	Ogbogbo 2 (22)	7.1064	6.8101	6 (27.3)	0 (0)	5 (22.7)	1 (4.5)
	Etutu (36)	7.2082	6.7921	5 (13.9)	1 (2.8)	4 (11.1)	0 (0)
	Ofuloko (20)	7.2163	6.7811	7 (3.2)	1 (5.0)	6 (30.0)	0 (0)
	Ujagba (9)	7.2082	6.7695	2 (22.2)	0 (0)	0 (0)	2 (22.2)
Ofu	Ejule 1 (25)	7.3604	7.0940	4 (16.0)	1 (4.0)	3 (12.0)	1 (4.0)
	Alome (22)	7.3385	7.0570	6 (27.3)	0 (0)	6 (27.3)	0 (0)
	Ejule (40)	7.3609	7.0985	16 (40.0)	14 (35.0)	3 (7.5)	0 (0)
	Ikpokejo-Umomi (20)	7.3435	7.0308	2 (10.0)	0 (0)	2 (10.0)	0 (0)
	Ofakaga (30)	7.4103	7.1250	4 (13.3)	2 (6.7)	2 (6.7)	0 (0)
Olamaboro	Ogugu 1 (35)	7.1553	7.4750	1 (2.9)	1 (2.9)	0 (0)	0 (0)
	Ogugu (36)	7.1518	7.4823	3 (8.3)	1 (2.8)	2 (5.6)	0 (0)
	Okpo (39)	7.2153	7.5570	8 (20.5)	2 (5.1)	4 (10.3)	1 (2.6)
	Ugbamaka-Igah (24)	7.2404	7.4688	5 (20.8)	0 (0)	5 (20.8)	0 (0)
	Igah-Ikeje (20)	7.1959	7.5296	4 (20.0)	0 (0)	4 (20.0)	0 (0)
Omala	Abejukolo (40)	7.8688	7.5061	9 (22.5)	0 (0)	9 (22.5)	0 (0)
	Opada (19)	7.8386	7.4853	7 (36.8)	0 (0)	7 (36.8)	0 (0)
	Agbenema-Ife (40)	7.8070	7.4533	20 (50.0)	0 (0)	20 (50.0)	0 (0)
	Abejukolo (40)	7.8644	7.5074	12 (30.0)	1 (2.5)	11 (27.5)	0 (0)
	Ajiyolo-Ife (23)	7.7637	7.4399	0 (0)	0 (0)	0 (0)	0 (0)
	Overall (1295)			222 (17.1)	56 (4.3)	164 (12.7)	14 (1.1)

n – Number examined.

### Spatial analysis of STHs

**Figure 1:**
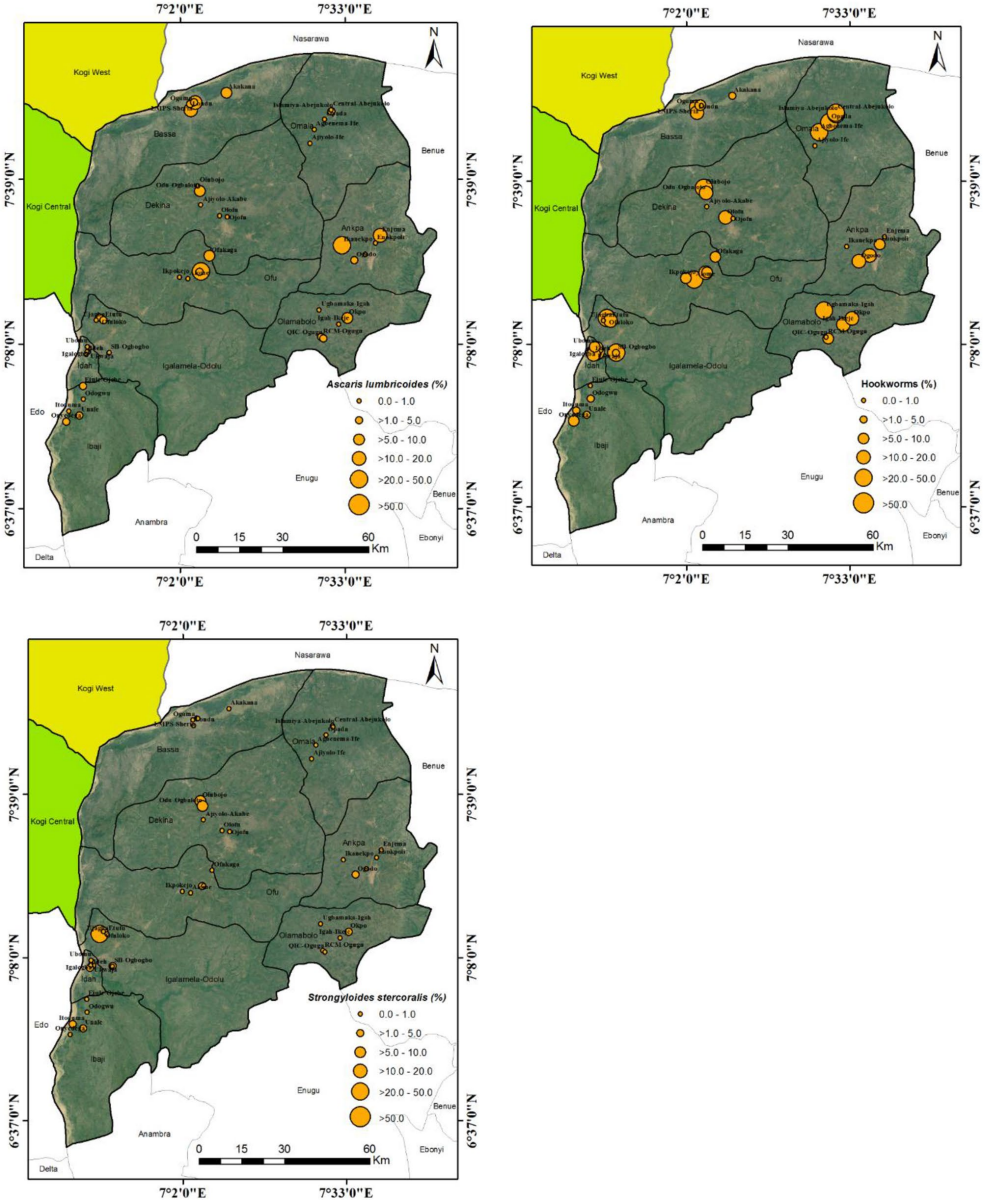
Spatial Distribution of STHs in Communities of Kogi East, North Central Nigeria. Source of Satellite Imagery: Image Google Earth: Landsat/Copernicus (Data SIO, NOAA, U.S. Navy, NGA, GEBCO. Maps were visualized on ArcMap 10.1. https://www.google.com/maps/place/Kogi/@7.3195959,7.2632804,189324m/data=!3m1!1e3!4m5!3m4!1s0x104f41e9d61f12dd:0xbdc9f94f2d58aafd!8m2!3d7.7337325!4d6.6905836.

**Figure 2:**
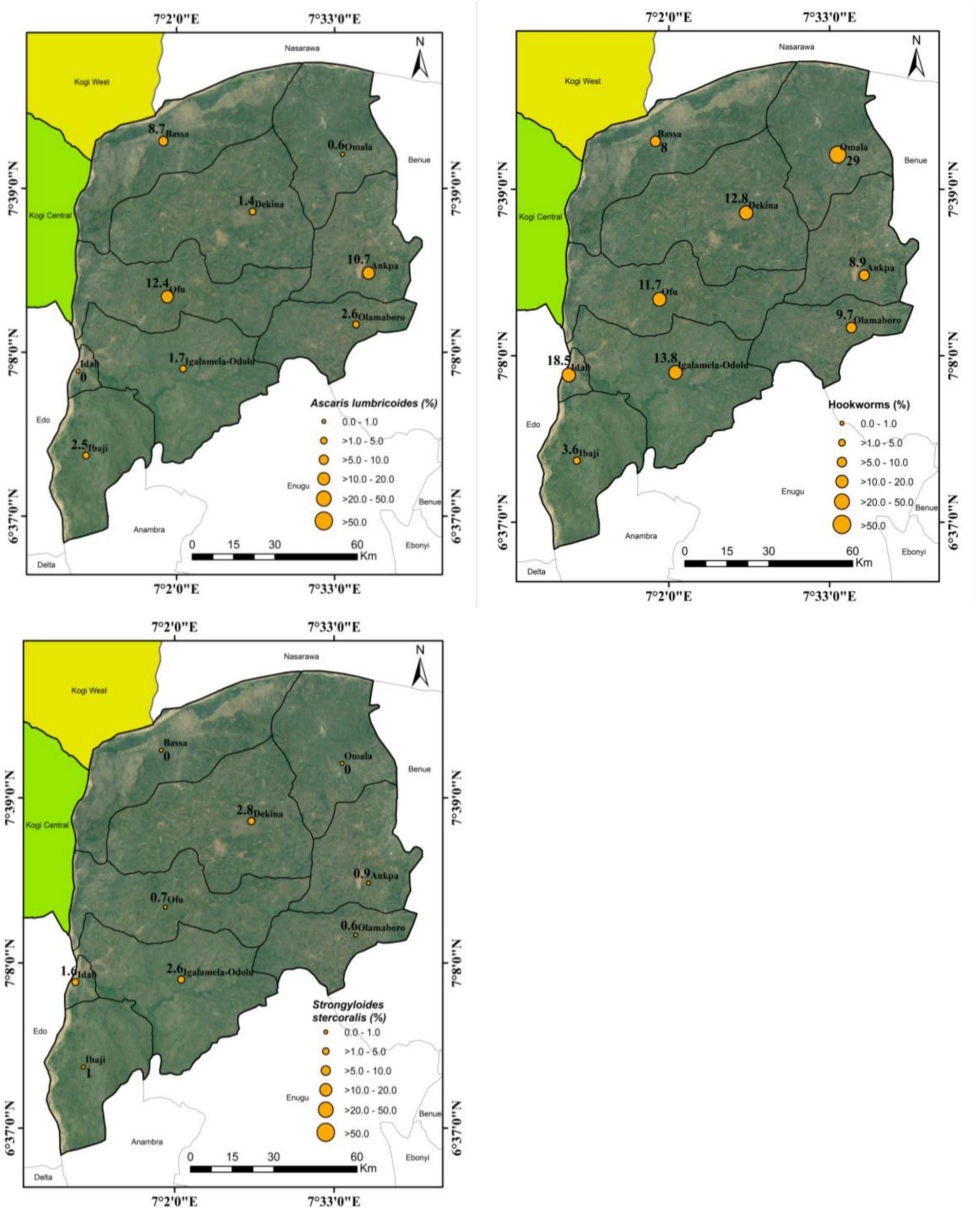
Spatial Distribution of STHs in Local Government Areas of Kogi East, North Central Nigeria. Source of Satellite Imagery: Image Google Earth: Landsat/Copernicus (Data SIO, NOAA, U.S. Navy, NGA, GEBCO. Maps were visualized on ArcMap 10.1. https://www.google.com/maps/place/Kogi/@7.3195959,7.2632804,189324m/data=!3m1!1e3!4m5!3m4!1s0x104f41e9d61f12dd:0xbdc9f94f2d58aafd!8m2!3d7.7337325!4d6.6905836.

Co-ordinate of schools sampled and the mean prevalence of each parasites from the baseline study for *A. lumbricoides*, Hookworms and *S. stercoralis* were computed in Microsoft Excel version 2013 and converted to comma delimited file (.csv). These files were further converted from text files to shapefiles using DIVA-GIS version 7.5.0 and were geo-referenced on the map of Kogi East, Nigeria. The prevalence of these parasites were categorized; 0.0–1.0, > 1.0–5.0, > 5.0–10.0, > 10.0–20.0, > 20.0–50.0 and > 50.0 on the map (Figs. 1 and 2).

### Environmental data collection

#### Climatic and elevation variables

Remotely sensed environmental data for altitude, temperature and precipitation were obtained from Worldclim database^30^. The climatic variables such as temperature and precipitation are at global and meso scales and topographic variables such as elevation and aspect likely affect species distributions at meso and topo-scales^31^. Hence, the use of the climatic and topographic variables in the prediction of distributions of soil transmitted helminths in Kogi East, Nigeria. Also, temperature was considered in the analysis because *A. lumbricoides*, hookworms and *S. stercoralis* have thermal thresholds of 38 °C, 40 °C and 40 °C respectively outside of which the survival of the infective stages in the soil decline^32,33^.

In this study, a total of 19 bioclimatic factors of present climate for Nigeria were downloaded at 1 km spatial resolution (Table 2) from Worldclim database^30^ and were used in the prediction of soil transmitted helminths distribution in Kogi East. Elevation data derived from the Shuttle Radar Topography Mission (SRTM) (aggregated to 30 arc-seconds, "1 km") were also downloaded from WorldClim database^30^.

**Table 2:** Characteristics of Environmental Variables Used in Predicting the Distribution of STHs in Nigeria.

Variable	Units	Spatial resolution	Data source	Accessible at
Elevation	M	1 km	Shuttle Radar Topography Mission (SRTM)	http://www.diva-gis.org/gdata
Bioclimatic variables (BIO 1 to BIO19) BIO1—Annual Mean Temperature BIO2—Mean Diurnal Range BIO3 – Isothermality BIO4—Temperature Seasonality BIO5—Maximum Temperature of Warmest Month BIO6—Mini Temperature of the Coldest Month BIO7—Temperature Annual Range BIO8—Mean Temperature of Wettest Quarter BIO9—Mean Temperature of Driest Quarter BIO10—Mean Temperature of Warmest Quarter BIO11—Mean Temperature of Coldest Quarter BIO12—Annual Precipitation BIO13—Precipitation of Wettest Month BIO14—Precipitation of Driest Month BIO15—Precipitation Seasonality BIO16—Precipitation of Wettest Quarter BIO17—Precipitation of Driest Quarter BIO18—Precipitation of Warmest Quarter and BIO19—Precipitation of Coldest Quarter	Temperature (Kelvin) Precipitation (mm)	1 km	WorldClimate	(WCD, 2004)
Soil organic carbon at the depth of 0–5 cm	Measure by either wet oxidation or dry combustion at 900 C in g/kg	250 m	International Soil Reference Centre (ISRIC)	(ISRIC, 2012)
pH of the soil at the depth of 0–5 cm	–	250 m	International Soil Reference Centre (ISRIC)	(ISRIC, 2012)
Soil clay content at the depth of 0–5 cm	Measure in g/100 g (w%)	250 m	International Soil Reference Centre (ISRIC)	(ISRIC, 2012)
Root zone moisture content aggregated at 30 cm (water retention capacity)	Measured in V%	1 km	International Soil Reference Centre (ISRIC)	(ISRIC, 2012)

#### Edaphic variable

The influence of edaphic factors on the distribution of STHs have been reported by several researchers globally^34–36^ as important factors in the biology of STH parasites. In view of this, data for soil pH, soil moisture content, soil organic carbon and soil clay content for Africa continent were downloaded from International Soil Reference Centre (ISRIC) soil database as spatial layers (Table 2)^37^.

### File conversions and resampling

The 19 bioclimatic factors downloaded from WorldClim data are in geographic coordinates of latitudes and longitudes which comes as .bil files were extracted into a folder. These data were transformed into predefined geographic coordinate system (GCS_WGS_1984), this projection was done on ArcMap 10.1 and were converted to asci files on DIVA-GIS 7.5. These files were transferred back to ArcMap and assigned a projected coordinate system of Universal Transverse Mercator (UTM) Zone 32 N (Nigeria is located on UTM Zone 31, 32 and 33). Also, the edaphic factors obtained were also assigned a projected coordinate system. The projected raster files (i.e. climatic, elevation and edaphic) were all clipped into a layer using the administrative boundary map of the study area, this was downloaded on DIVA-GIS database^38^.

Prior to modelling, all variables were resampled from their native resolution to a common resolution of 1 km spatial resolution using the nearest neighbour technique on ArcMap 10.1 to enable overlaying of variables. The resampled raster files were converted to float files on ArcMap 10.1 and transferred to DIVA-GIS 7.5. Float files were converted to grid files and then to asci files on DIVA-GIS 7.5 and were used on MaxEnt tool for modelling the distribution of STHs in Kogi East.

### Ecological niche modelling

The potential distribution of STHs were modelled using maximum entropy (MaxEnt) software version 3.3.3k^39^. MaxEnt uses environmental data at occurrence and background locations to predict the distribution of a species across a landscape^31,40^. This modelling tool was selected based on the reasons of Sarma et al.^41^, they stated that this tool allows the use of presence only datasets and model robustness is hardly influenced by small sample sizes. It has been shown to be one of the top performing modelling tools^42^.

Probability of presence of each of the STH was estimated by MaxEnt using the prevalence of each of the STH parasites obtained for 45 sampled communities in the 9 LGAs of Kogi East during the district-wide survey carried out in 2018^25^ served as the presence records to generate background points were used^41^. Regularization of the prevalence was performed to control over-fitting. This modelling tool uses five different features to perform its statistics; linear, quadratic, product, threshold and hinge features to produce a geographical distribution of species within a define area. The MaxEnt produces a logistic output format used in the production of a continuous map that provides a visualization with an estimated probability of species between 0 and 1. This map distinguish areas of high and low risk for STH infections^41^.

The 19 bioclimatic factors, elevation data and the edaphic factors obtained were used for the ecological niche modelling. The level of significance of contribution of the altitude and 19 bioclimatic factors was used to calculate the area under the receiver operating characteristics curve (AUC) was used to evaluate the model performance. The AUC values varies from 0.5 to 1.0; an AUC value of 0.5 indicates that model predictions are not better than random, values < 0.5 are worse than random, 0.5–0.7 signifies poor performance, 0.7–0.9 signifies reasonable/moderate performance and > 0.9 indicates high model performance^43^.

Model validation was performed as follows^41^, using the ‘sub-sampling’ procedure in MaxEnt. 75% of the parasites prevalence data were used for model calibration and the remaining 25% for model validation. Ten replicates were run and average AUC values for training and test datasets were calculated. Maximum iterations were set at 5000. Sensitivity, which is also named the true positive rate, can measure the ability to correctly identify areas infected. Its value equals the rate of true positive and the sum value of true positive and false negative. Specificity, which is also named the true negative rate, can measure the ability to correctly identify areas uninfected. Its value equals the rate of true negative and the sum value of false positive and true negative.

### Ethics approval

This study follows guidelines for the care and use of experimental animals established by the Animal Care and Use Committee of the Ahmadu Bello University, Zaria for the purpose of control and supervision of experiments on animals and ethical permission for the study was obtained from the ethical Board of Kogi State Ministry of Health, Lokoja with reference number: MOH/KGS/1376/1/82.

## Results

### Predicted Risk of *A. lumbricoides*, hookworms and *S. stercoralis* Infections

**Figure 3:**
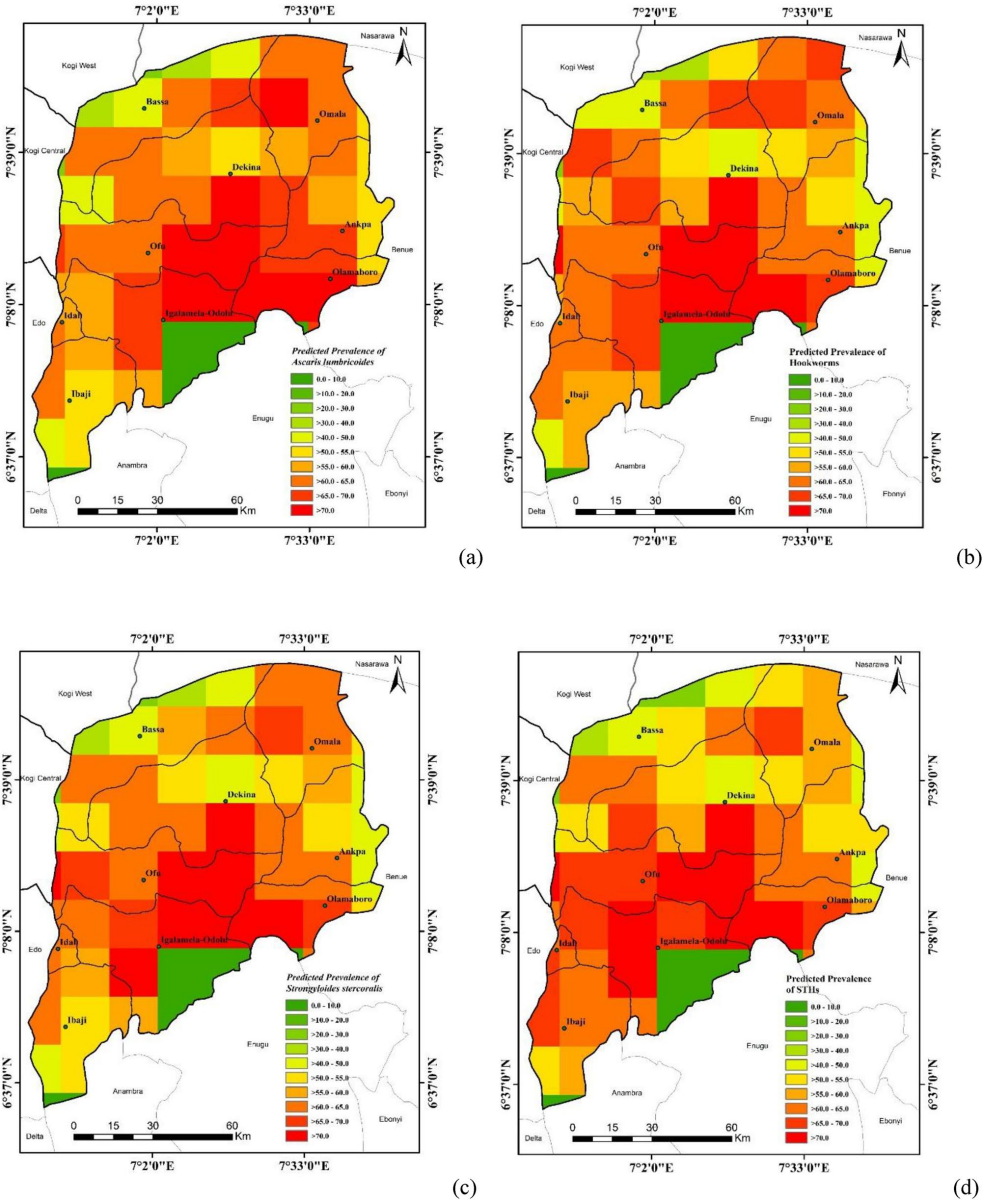
High Resolution Risk Maps of STHs in Kogi East, North Central Nigeria **a** *A. lumbricoides* **b** Hookworms **c** *S. stercoralis* **d** STHs Combined. Maps were visualized on ArcMap 10.1.

The predicted high risk areas of *A. lumbricoides* were the central part of Kogi East i.e. Southern part of Dekina LGA, Eastern part of Ofu LGA, northern part of Igalamela-Odolu LGA, the west-Southern part of Olamaboro LGA and the Eastern part of Omala LGA in Northern Kogi East with probability of 0.8 to 1.00. The extreme northern part of Kogi East (upper part of Bassa LGA) and South Eastern part of Igalamela-Odolu LGA and extreme part of Southern Ibaji LGA were areas in the district with low risk of infections with *A. lumbricoides* with probability of 0.0 – 0.3 (Fig. 3a).

For hookworms, there is an overlap in the risk areas similar to what was observed for *A. lumbricoides* except in the northern part of Omala. The high risk areas for hookworm infections lies in the Central part of Kogi East. The extreme eastern part of Ankpa LGA, extreme northern part of Bassa LGA, extreme part of Southern Ibaji and Eastern part of Igalamela Odolu LGA are areas that fell within the low risk areas (Fig. 3b). Probability ranges from 0.0 to 0.3 for low risk areas and 0.7–1.00 for high risk areas.

The high risk areas for hookworms were the same for *S. stercoralis* as well as the low risk areas (Fig. 3c).

The combined predicted map for the STHs (Fig. 3d) indicates high risk areas in Dekina, Igalamela-Odolu, Idah, Ofu and Olamaboro LGAs with patches in Omala and Ibaji LGAs.

### Model performance and influencing factors

The mean percent contribution and permutation importance are two factors used to assess the effectiveness of variables used in the modelling of STH in Kogi East. A total of 24 factors were assessed, categorized into elevation (altitude), climatic and edaphic variables. Of these variables assessed, mean diurnal range (BIO2) was the factor with the highest importance and contribution in determining the distribution of STHs in Kogi East. It had the highest PC and PI of 36.7% and 59.9%, 39.7% and 64.2%, and 36.5% and 57.5% for *A. lumbricoides*, hookworms and *S. stercoralis* respectively (Table 3).

**Table 3:** Average Percent Contribution and Average Permutation Importance (API) of STHs Infection Distribution Models.

*Ascaris lumbricoides*				Hookworms				*Strongyloides stercoralis*			
Factor	PC	Factor	PI	Factor	PC	Factor	PI	Factor	PC	Factor	PI
Bio_2	36.7	Bio_2	59.9	Bio_2	39.7	Bio_2	64.2	Bio_2	36.5	Bio_2	57.5
Bio_18	23.7	Bio_16	14.6	Bio_18	23.4	Bio_16	15	Bio_18	25.1	Bio_16	12.2
Bio_7	15.3	Bio_13	9	soil_clay	15.3	Bio_13	9.1	Bio_7	16.3	Bio_13	8.3
soil_clay	14.6	Bio_18	7.3	Bio_7	14.9	soil_clay	7.4	soil_clay	14.5	soil_clay	7.6
Bio_5	2.1	soil_clay	3.8	Bio_13	1.7	Bio_18	2.3	Bio_5	1.6	Bio_7	7.1
Bio_13	1.9	Bio_7	2.3	Bio_5	1	Bio_17	1.2	Bio_13	1.4	Bio_18	5.7
Ph	1.8	Bio_17	2.1	ph	0.9	Bio_7	0.5	ph	1.1	Bio_17	1.2
Bio_17	1.1	Ph	0.4	Bio_16	0.8	ph	0.3	WaretCap	0.9	ph	0.3
WaretCap	1.1	WaretCap	0.4	Bio_17	0.8			Bio_17	0.7	Bio_5	0.1
Bio_16	0.7	Bio_5	0.2	Bio_6	0.4				Bio_15	0.6	
Bio_4	0.4			Bio_15	0.3				Bio_16	0.6	
Bio_15	0.3			Bio_4	0.2				Bio_6	0.5	
Bio_6	0.3			Bio_19	0.2				Bio_4	0.2	
				SOC	0.2						
				WaretCap	0.2						
Total	100		100		100		100			100	100

PC—Percent Contribution, PI—Permutation Importance.

BIO2—Mean Diurnal Range, BIO4—Temperature Seasonality, BIO5—Maximum Temperature of Warmest Month, BIO6—Mini Temperature of the Coldest Month., BIO7—Temperature Annual Range, BIO13—Precipitation of Wettest Month, BIO15—Precipitation Seasonality, BIO16—Precipitation of Wettest Quarter, BIO17—Precipitation of Driest Quarter, BIO18—Precipitation of Warmest Quarter and BIO19—Precipitation of Coldest Quarter, soil_clay—Soil Clay, ph—pH, WaretCap—Water Retention Capacity.

Precipitation of the warmest quarter (BIO18) was the second ranked in terms of PC for the three parasites with 23.7%, 23.4% and 25.1% for *A. lumbricoides*, hookworms and *S. stercoralis* respectively while precipitation of wettest quarter (BIO16) was the second ranked in terms of PI with 14.6%, 15.0% and 12.2% for *A. lumbricoides*, hookworms and *S. stercoralis*. Soil clay content and temperature annual range (BIO7) interchangeably ranked third and fourth in their PC in determining the distribution of these parasites; *A. lumbricoides* (3rd—BIO7: 15.3%, 4th—Soil clay: 14.6% and 5th – BIO5: 2.1%), hookworms (3rd—soil clay: 15.3%, 4th—BIO7: 14.9%, 5th—BIO13: 1.7%) and *S. stercoralis* (3rd—BIO7: 16.3%, 4th soil clay: 14.5%, 5th—BIO5: 1.6%) (Table 3).

For the PI; BIO13 (9.0%), BIO18 (7.3%) and soil clay (3.8%) ranked third, fourth and fifth in determining the distribution of *A. lumbricoides*; BIO13 (9.1%), soil clay (7.4%) and BIO18 (2.3%) ranked third, fourth and fifth in determining the distribution of hookworms while BIO13 (8.3%), soil clay (7.6%) and BIO7 (7.1%) ranked third, fourth and fifth in determining the distribution of *S. stercoralis* (Table 3).

**Figure 4:**
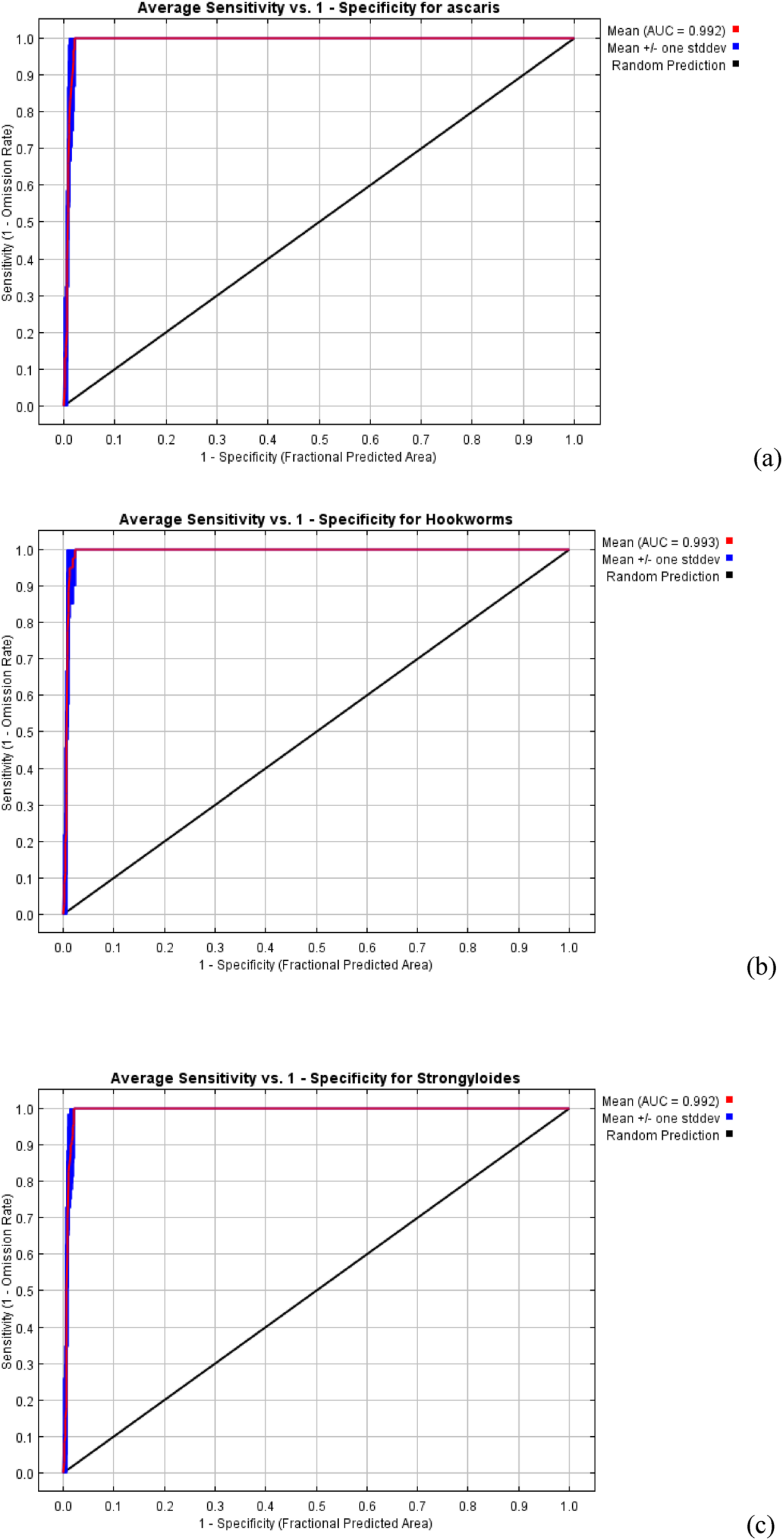
Receiver Operating Characteristics (ROC) curves for **a** *A. lumbricoides* **b** Hookworms and **c** *S. stercoralis*. Red line indicates the mean value for 10 MaxEnt replicate runs and blue indicates the standard deviation.

The receiver operating characteristics (ROC) curves were obtained as an average of 10 replicates run on MaxEnt, specificity and sensitivity for each parasite was calculated as presented in Fig. 4a,b,c. The average and standard deviation of the Area Under the Curve (AUC) for the 10 replicate runs were 0.992, 0.993, and 0.992 for *A. lumbricoides*, hookworms and *S. stercoralis* respectively. These values are indication of excellent performance of the modelling software as an AUC value for greater than 0.80 showed higher sensitivity and specificity for the presence of these parasites.

The modelling result of STHs in Kogi East, Nigeria revealed that the high risk areas lies in the Central part of Kogi East District in the LGAs of Dekina, Ofu, Olamaboro and Igalamela-Odolu.

## Discussion

This study revealed that both climatic and edaphic factors influence the distribution of STHs at varying degree. In this study, mean diurnal temperature range (BIO2), temperature annual range (BIO7), maximum temperature of the warmest month (BIO5), precipitation of the wettest quarter (BIO16)], and soil clay contents were the top five most important factors responsible for their distribution out of the 24 factors investigated. A study carried out in Zimbabwe^35^ on the inclusion of edaphic factors in enhancing the distribution of STHs reported that the inclusion of edaphic factors alongside other environmental variables enhance the performance of the predictive models used in determining the distribution of these parasites.

Some studies carried out in Bolivia^34^, China^44^ and Nigeria^26^ predicted the distribution of these parasites using only environmental variables might have either underestimated or overestimated the distribution. Comparison of this study with a previous study in Nigeria^27^ in which only environmental variables were used revealed an improvement in the model i.e. they observed an AUC of 0.948 compared to an AUC of 0.992 in this current study (4.44% increase in model performance). Therefore, inclusion of edaphic variable proved to be important in determining the distribution of STHs especially factors such as soil clay, pH and soil moisture (or soil water retention capacity).

Soil organic carbon was very important in determining the distribution of hookworms. Similar observation was reported in the Zimbabwe^35^, they stated that soil organic carbon is among the highly important factor in determining the distribution of hookworms, this was due to the ecology of hookworms as a parasite that feeds on organic matter. This observation is similar also to a study on Sandy soil in KwaZulu-Natal, South Africa^36^.

For the climatic variables, mean diurnal range (BIO2), precipitation of the wettest quarter (BIO18), precipitation of wettest quarter (BIO16), Temperature annual range (BIO7) and precipitation of wettest month (BIO13) were the factors of most relative importance in the determining the distribution of STHs in Kogi East. An earlier study in Nigeria reported^27^ that precipitation of the wettest month was the most important factor in STHs distribution in Nigeria. This result is also in line with previous study^14^ on moisture requirement of eggs of parasitic ascarids in mammals. A study in Kebbi State, Nigeria^45^ stated the significance of moisture and warm conditions in enhancing the embryonation of the eggs of parasitic helminths.

Mean diurnal temperature range (BIO2), temperature annual range (BIO7), maximum temperature of the warmest month (BIO5), precipitation of the wettest quarter (BIO16)], and soil clay contents were the five factors that exerted most significant influence on the geospatial distribution of STHs in Kogi East, Nigeria.

Sanitation is a crucial environmental factor in eliminating the overall rates of STH infection. However, the higher cost of proper sanitation methods compared to other intervention limit its implementation in many communities particularly where resources are limited^46^. This factor might be responsible for lack of data on sanitation from our study area since they are rural communities with limited resources. This is a limitation of this study. Further research incorporating sanitation parameters to evaluate its effect on the distribution of STHs should be conducted.

## Conclusions

Mean diurnal range (BIO2), precipitation of the wettest quarter (BIO16), soil clay content, temperature annual range (BIO7) and maximum temperature of the warmest month (BIO5) are the five most important environmental risk factors that plays significant role in the spatial distribution of STHs in Kogi East, North Central Nigeria. Southern part of Dekina LGA, Eastern part of Ofu LGA, northern part of Igalamela-Odolu LGA, the west-Southern part of Olamaboro LGA and the Eastern part of Omala LGA were areas at high risk of infection with STHs. Policies designed on the monitoring of the distribution of chemotherapy should be reviewed properly by selecting and considering high-risk communities, this will allow maximum achievement of control strategy.

## Data Availability

The data sets in this study are available from the corresponding author on reasonable request.

## Abbreviations

NTDs: Neglected tropical diseases
LGA: Local government area
STHs: Soil-transmitted helminths
STH: Soil-transmitted helminthiasis
GIS: Geographic information systems (GIS)
RS: Remote sensing
SDM: Species distribution models
GPS: Global positioning system
UTM: Universal transverse mercator
ISRIC: International soil reference centre
AUC: Area under curve
ROC: Receiver operating characteristics
PI: Permutation importance
PC: Percent contribution
